# Supplementary material for: Incidence and patterns of adverse drug reactions among adult patients hospitalized in the University of Gondar comprehensive specialized hospital: A prospective observational follow-up study
Source: PLoS One. 2023 Feb 24;18(2):e0282096. doi: 10.1371/journal.pone.0282096 (PMC9955665; doi:10.1371/journal.pone.0282096)
Supplement: S1 File — (PDF) [file pone.0282096.s001.pdf]

Incidences, patterns and associated factors of adverse drug reactions among adult patients hospitalized at medical ward of University of Gondar: Observational Study by Ashenafi et a1.

Data collection instrument and participants informed consent

Dear participant,

You are randomly selected to participate in this study. The aim of the study is to assess the incidences, patterns and associated factors of adverse drug reactions among adult patients hospitalized at medical ward of University of Gondar Comprehensive specialized hospital; the questioner comprises questions regarding your sociodemographic, clinical, and medication-related information related to your medical conditions and current admissions. We would like to get your consent to participate in this study. The duration and collection of the data will be on a daily bases until you will discharged out. Meanwhile, there are some interviews regarding medication related adverse effects and we will review your medical records for clinical and laboratory parameters. The information we obtain will remain strictly confidential. Your answer and name will never be revealed. We assure you that it is totally voluntary. You have not gained any fee from your participation or suffered any harm because of your refusal. But your participation is greatly appreciated for the completion of the study. If you agree to participate, we will interview you. You wish to refuse or withdraw at any point in the study.

Do you agree to participate in this study? 1. Yes Signature----------------------------

Thank You!!!

Part I: Sociodemographic characteristics of the study participants

**Variables**	**Category**
Card Number	
Date of admission	
Sex	1. Male 2. Female
Age (years)	
Residency	1. Urban 2. Rural
Body weight (Kg)	-
Body height (M)	-
Educational status	1. Unable to read and write 3. Secondary school<br>2. Primary school 4. College and above
Marital status	1. Single (never married) 2. Married 3. Divorced 4. Widowed
Source of Healthcare coverage	1. Health insurance 3. Non-insurance organizations<br>2. Out of pocket
Work/Occupation	1. Government employee 3. Farmer 5. Unemployed<br>2. Self-employee/merchant 4. Student 6. Others/specify

Average monthly household income (Eth.birr)	1. < 1500 2. 1500-2999 3. 3000-4999 4. ≥ 5000
Smoking status	1. Currently smoking 2. Previously smoker 3. Nonsmoker at all
Alcohol use habits	1.Yes 2. No
Physical activity	1. Sedentary (no) 2. Moderate 3. Vigorous

Part II: Clinical and medication related characteristics of the study participants

**Variables**	**Category**
Admission diagnosis (cause of admission)	
Number of previous admissions in the last 6 moths	
Type of illness	1.Noncomnicable 2. Communicable 3. Other /specify
If non communicable	1.Newly diagnosis 2. Chronic
If chronic, duration since diagnosis (years)	
If chronic, reason for current admission	1.Exacerbation 3. ADR 5. other/specify<br>2. Poor adherence 4. Complication
If ADR is reason of admission, describe name and characterize	
List of medications were taking	
Potential offending agent of ADR	
History of similar ADRs	
Preexisting comorbidity	1. Yes 2. No
Number of comorbidities	
Type (name) of comorbidity	-<br>-
Complication of initial diagnosis	1.Yes 2. No
Number of complications	
Type(name) of complication	
Experience of hospitalized infection	1. Yes 2. No
Using of lifestyle modification	1. Yes 2. No
Which type of lifestyle modification	1. Dietary 2. Physical activity 3. Dietary and physical activity
Medication on admission	-<br>-<br>-
Add medication during follow-up	-<br>-
Number of medications per patient	
**Adverse drug reactions**	
Experience of ADR after admission	1. Yes 2. No
If yes, describe name and characterize the signs and symptoms of ADR	
Possible offending agents caused for ADR	

Days/hrs. since initiation of offending agent to experience of ADR	
Management of ADR<br>Both pharmacological and non-pharmacological	
Is ADR reversed/cured?	1. Yes 2. No
If yes, time takes since experience to ADR reversed	
Severity of existing ADRs	
Total length of stay since admission to final patient outcome (in days)	
Date of final outcome recorded	

**Laboratory parameters**	Baseline	During follow-up	At the end of follow up
CBC: Hgb<br>Hct.			
RFT: Scr.<br>GFR			
LFT: GPT/SGPT<br>GOT/SGOT			
Blood glucose: RBG<br>FBG<br>HbA1c			
Electrolyte: Na^{+1}<br>K+1			
Lipid profiles: LDL<br>HDL<br>Total cholesterol (TC)<br>Total glyceride (TG)			

Part III: Naranjo Adverse Drug Reaction Probability Scale that can assess the likelihood of the ADRs related to exposed medications

Questions	Yes	No	Do not know	Score
1. Are there previous conclusive reports on this reaction?	+1	0	0	
2. 2. Did the adverse event appear after the suspected drug was administered?	+2	-1	0	
3. Did the adverse reaction improve when the drug was discontinued or a specific antagonist was administered?	+1	0	0	
4. 4. Did the adverse event reappear when the drug was re-administered?	+2	-1	0	
5. Are there alternative causes (other than the drug) that could on their own have caused the reaction?	-1	+2	0	
6. Did the reaction reappear when a placebo was given?	-1	+1	0	
7. Was the drug detected in blood (or other fluids) in concentrations known to be toxic?	+1	0	0	
8. Was the reaction more severe when the dose was increased or less severe when the dose was decreased?	+1	0	0	
9. Did the patient have a similar reaction to the same or similar drugs in any previous exposure?	+1	0	0	
10. Was the adverse event confirmed by any objective evidence?	+1	0	0	
**Total Score:**				

Modified from: Naranjo CA et al. A method for estimating the probability of adverse drug reactions. Clin Pharmacol Ther 1981; 30: 239245

Part IV: Severity of Adverse drug rection based on Hartwig Severity Assessment Scale, which assess the severity of the existing ADRs.

Incidences, patterns and associated factors of adverse drug reactions among adult patients hospitalized at medical ward of University of Gondar: Observational Study by Ashenafi et a1.

Level	Description of ADR's characteristics	Frequency	Percent
1	An ADR occurred but required no change in treatment with the suspected drug		
2	The ADR required that treatment with the suspected drug be held, discontinued, or otherwise changed. No antidote or other treatment requirement was required. No increase in hospital stays.		
3	The ADR required that treatment with the suspected drug be held, discontinued, or otherwise changed AND/OR an antidote or another treatment was required. No increase in hospital stays.		
4	Any Level 3 ADR which increases the length of stay by at least 1 day.		
5	Any level 4 ADR which requires intensive medical care.		
6	The ADR caused permanent harm to the patient		
7	The ADR which led to the death of the patient		

mild = level 1 and 2), moderate = level 3 and 4) and severe (level 5 and above)

Hartwig SC et al. Severity assessment in reporting adverse drug reactions. Am J Hosp Pharm. 1992;49(9):2229–32
